# The Effects of the Melatonin Treatment on the Oxidative Stress and Apoptosis in Diabetic Eye and Brain

**DOI:** 10.1100/2012/498489

**Published:** 2012-05-01

**Authors:** Tuğba Gürpınar, Nuran Ekerbiçer, Nazan Uysal, Turgay Barut, Figen Tarakçı, M. Ibrahim Tuglu

**Affiliations:** ^1^Department of Pharmacology, Faculty of Medicine, Celal Bayar University, 45030 Manisa, Turkey; ^2^Department of Physiology, Faculty of Medicine, Celal Bayar University, 45030 Manisa, Turkey; ^3^Department of Physiology, Faculty of Medicine, Dokuz Eylul University, 35340 Izmir, Turkey; ^4^Department of Histology and Embriology, Faculty of Medicine, Celal Bayar University, 45030 Manisa, Turkey; ^5^Department of Vocational School of Health Services, Celal Bayar University, 45030 Manisa, Turkey; ^6^Department of Histology, Faculty of Medicine, Celal Bayar University, 45030 Manisa, Turkey

## Abstract

Oxidative stress plays an important role in the development of complications in diabetes mellitus. Antioxidant therapy has been thought to decrease oxidative stress. The objective of the present study was to explore the effects of melatonin (MLT) on oxidative stress in diabetic rat eye and brain tissue by using immunohistochemical methods. Diabetes was induced by streptozotocin, (STZ, 55 mg/kg/i.p) in adult rats. MLT was given 10 mg/kg/i.p once a day for 2 weeks beginning from the sixth week. Six weeks later, rats were divided into three groups: control (CR), STZ-induced diabetic (STZ), and STZ-induced diabetic group received melatonin (STZ+MLT). Although no significant difference was observed with respect to antioxidant status, NOS activity tended to be higher in the untreated diabetic rats than in the treated rats. It was observed that MLT treatment improved the histopathological changes including apoptosis and oxidative stress in brain and eye in diabetic rat.

## 1. Introduction

Diabetes mellitus is a chronic disorder associated with hyperglycemia, hyperlipoproteinemia, and oxidative stress [1, 2]. Oxidative stress appears to be the most important pathogenic factor in underlying diabetic complications [1, 3]. Reactive oxygen species (ROS) can modulate cellular function, receptor signals, and immune responses in physiological conditions, but excessive production of ROS can mediate progressive endothelial damage through growth and migration of vascular smooth muscle and inflammatory cells, causing alteration of extracellular matrix and apoptosis of endothelial cells [4, 5]. ROS alter vascular tone by increasing concentration of cytosolic calcium and reducing the bioavailability of vascular NO relates to its rapid oxidative inactivation resulting decreased availability of nitric oxide [6, 7]. Recent investigations indicated that during oxidative stress elevation of ROS and reduction of superoxide dismutase were accompanied by induction of iNOS and increased NO-ROS reaction and also increased collagen, TGF-*β*1, plasminogen activator inhibitor, and apoptosis were found in diabetic tissues [7, 8].

Melatonin (N-acetyl-5-methoxytryptamine) is an endogenous neurohormone derived from tryptophan, that is, mainly released from the pineal gland [9–11]. Melatonin participates in a number of physiological processes like the reproduction regulation and circadian rhythms, at the same time is a well-known potent antioxidant and well toleratant upon its administration [12, 13]. Melatonin is an effective scavenger of different ROS, such as hydroxyl and peroxyl radicals cross all morphophysiological barriers, is distributed throughout all cells and also has a powerful capacity to scavenge free radicals and prevents tissue damage [9, 14]. In a recent study, melatonin was showed to decrease oxidative stress in streptozotocin-induced diabetic rats [15]. In another study, Sáenz et al. indicates that melatonin could be a potent inhibitor of the retinal nitridergic pathway [16]. Therefore, we designed this study to investigate the effects of MLT treatment on the eye and brain of the diabetic rat using immunological and histological methods related to apoptosis and oxidative stress.

## 2. Materials and Methods

Eighteen male Wistar rats (250–350 g) had access to laboratory food and water *ad libitum. *They were housed in cages under standard laboratory conditions (light period between 07:00–19:00 h; 21±2°C; relative humidity 55%). This study was approved by the Institutional Animal Care Ethics Committee of Celal Bayar University, Turkey. Diabetes was induced by a single intraperitoneal injection of streptozotocin (STZ, Sigma, St. Louis, MO; 55 mg/kg, freshly dissolved in distilled water). Twenty-four hours after STZ treatment, development of diabetes in the experimental groups was confirmed by measuring blood glucose levels in blood samples taken from the tail vein; rats with blood glucose levels 250 mg/dL were considered diabetic [17]. Six weeks after STZ treatment, rats were treated with Melatonin (MLT) i.p at a dose of 10 mg/kg/day for two weeks. Similar doses and durations of melatonin administrations are available in the literature [15, 18].

 Rats that died, got sick, or did not develop diabetes were excluded from the study. As a result, the study was completed with six rats in each group for a total of 18 rats. Animals were assigned randomly to one of three groups as follows: (1) control group (CT, *n* = 6), (2) STZ-induced diabetic group (STZ-DM, *n* = 6), and (3) ATV-treated STZ-induced diabetic group (STZ+MLT, *n* = 6).

For immunohistochemical staining, sections were incubated at 60°C overnight and then in xylene for 30 min. After washing with a decreasing series of ethanol, sections were washed with distilled water and phosphate-buffered saline (PBS) for 10 min. Sections were then treated with 2% trypsin at 37°C for 15 min. After washing with PBS, they were incubated in a solution of 3% H_2_O_2_ for 15 min to inhibit endogenous peroxidase activity. Then sections were washed with PBS and incubated for 18 h at +4°C with primary antibodies: a monoclonal anti-eNOS (rabbit Pab, RB-1711-P1, Neomarkers, Fremont, CA, USA), anti-iNOS (rabbit Pab, RB-1605-P, Neomarkers, Fremont, CA, USA), and antibodies against TGF-*β*1 (Santa Cruz Biotechnology, SC146). Afterwards, sections were washed 3 times for 5 min each with PBS, followed by incubation with biotinylated goat IgG anti-rabbit IgG and then with streptavidin conjugated to horse-radish peroxidase for 30 min each (Dako LSAB 2 kit, Peroxidase). After washing, 3 times for 5 min with PBS, sections were incubated DAB (Dako) for 5 min to stain immunolabelling and then with Mayer's hematoxylin. Sections were covered with mounting medium and were analyzed light microscopically with a BX 40 microscope (Olympus, Tokyo, Japan). Control samples were processed in an identical manner, but primary antibody was omitted. Two observers blinded to clinical information evaluated the staining scores independently. Detection of the apoptotic cell death *in situ* using as TUNEL method was used for programme cell death mechanism. Fragmentation of the DNA in the nucleus is one of the first morphological changes of the apoptotic process and can be detected in histological sections using a terminal deoxynucleotidyltransferase-biotin nick end-labeling method (TUNEL) performed with a commercial kit (DeadEnd Colorimetric TUNEL system, Promega G7130) according to the manufacturer's instructions. Briefly, after proteinase K treatment for 10 min, the sections were incubated at 370°C with TdT for 60 min. As negative staining control for TUNEL, TdT was omitted during the tailing of reactions.

The data were expressed as mean ± standard deviation (SD). The data were analysed using repeated measures of variance. Tukey Kramer multiple comparisons test was used to test for differences among means when ANOVA indicated a significant *P* value (*P* < 0.05).

## 3. Results

**Figure 1:**
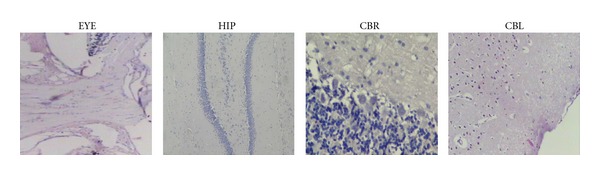
Histopathologic image of eye (EYE), hippocampus (HIP), cerebrum (CBR), and cerebellum (CBL) after MLT treatment. (H&E ×200).

**Figure 2:**
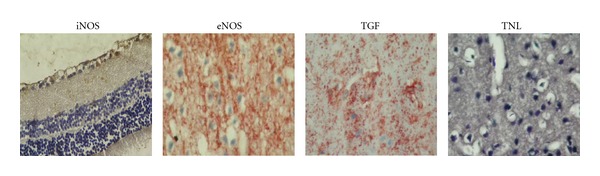
Immunohistochemical image of eye (EYE), hippocampus (HIP), cerebrum (CBR), and cerebellum (CBL) after MLT treatment. Because of the similarity of histologic samples, an image was given for each tissue ×200.

**Figure 3:**
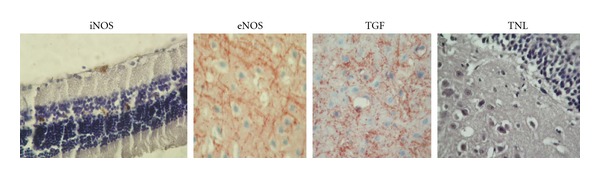
Histolopathology of TUNEL images of eye (EYE), hippocampus (HIP), cerebrum (CBR), and cerebellum (CBL) after MLT treatment. Because of the similarity of histologic samples, an image was given for each tissue ×200.

**Figure 4:**
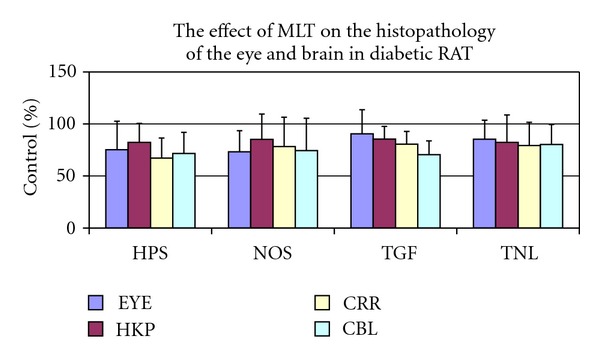
Morphometric analyse of the effect of the MLT treatment on the damage on eye (EYE), hippocampus (HIP), cerebrum (CBR), and cerebellum (CBL). Data were expressed as % comparisons with control values mean ± standard deviation (SD).

No obvious motor or sensory deficits were observed in any of the subjects before the experiment. There was a significant increase in fasting blood glucose levels in STZ (350 ± 25) diabetic rats compared with the CT (90 ± 18) group. There was no statistically significant difference between STZ and STZ+MLT groups (319 ± 35). In addition, there were no pathologic findings observed in the optic nerve, whereas endothelial damage was stated in the vessels. In the brain samples, hippocampus, cortex, and cerebellum have also shown endothelial damage (Figures 1 and 2). There were no significant pathologic differences in histological morphometry (Figure 4) which is used in revealing cell degeneration and death and TUNEL (Figure 3) which is used to evaluate apoptosis. TGF-*β*1 was used to detect damage in vascular tissues, and iNOS, and eNOS immunoreactivities were used to determine oxidative stress. eNOS reactivity was found to be more than iNOS reactivity, however, there was minimal increase stated in diabetic rats. MLT treatment causes decrease in all findings but it was not statistically significant. 

## 4. Discussion

In our study, we observed no significant edema or damage in the retina and brain in diabetic rats. Basal oxidative stress was determined in the tissue samples, however, it was not at level to cause damage. Observed minimal damage found to be related in apoptosis and oxidative stress in vascular tissue. MLT treatment reduced the damage and suggested to affect histopathologic changes by reducing oxidative stress.

Oxidative stress has been implicated in the major complications of diabetes mellitus [3]. Decreased antioxidant capacity and/or increased production of ROS are the two common mechanisms that lead to increased oxidative stress in diabetes mellitus, therefore tissue damage is facilitated Not only lipids but also proteins, carbohydrates, and nucleic acids are affected by alteration of the oxidant and antioxidant systems [2, 3]. Our results were well matched to the previous studies that shown oxidative stress in streptozotocin-induced diabetic rats [15]. It was suggested that vascular complications became more important in this media and verified by both experimental and clinical studies. Lower endogeneous antioxidants and elevated lipid peroxidation levels are risk factors for the development of macro- and microvascular diabetic complications such as retinopathy, neuropathy, nephropathy, cataracts, and atherosclerosis [1, 2]. As oxidative stress is the main cause of diabetic complications administration of antioxidants appears one of the most reasonable therapeutic approaches. Some studies showed that diabetic complications may be reduced by antioxidant therapies and different antioxidants, including vitamins C and E, lipoic acid, and L-Carnitin, in a variety of experimental animal models of diabetes. They are also able to improve insulin and glucose levels and reduce micro- and macrovascular dysfunction [18, 19].

Melatonin, whose antioxidant properties are well documented, is attracting increased attention in recent years and is known to reduce oxidative stress [19, 20]. Melatonin has produced mainly in pineal gland which is considered to be one of the most potent antioxidant agents which have negligible toxicity even in the very high doses [9, 19]. Jaworek et al. [21] have shown that melatonin counteracts the increase in the ROS-induced lipid peroxidation and preserves, at least in part, the activity of key antioxidizing enzymes, such as superoxide dismutase, and an important role in prevention of gastric and pancreatic damage. In another study, melatonin was found to reduce apoptosis and necrosis induced by ischemia/reperfusion injury of the pancreas [22]. Taken together with the results from the other studies, this study showed that the relationship between oxidative stress and cell damage supported by not only biochemical but also histological findings. Also in agreement with the other reports, melatonin treatment does not attenuate diabetic hyperglycaemia, it effectively ameliorates oxidative stress accompanying diabetes. In view of our findings, melatonin seems to be a promising agent for diabetes therapy. Another interesting finding according to our study that the tissue response to oxidative stress evolves more quickly in the vascular tissue especially in the endothelial cells and apoptosis is thought to be a natural end of this situation. Any change in vascular function results in chronic endoneurial vascular damage, which in turn results in the neurodegeneration seen in diabetic patients. Piotrowski found the activity of caspase-3 (CPP32) increased in hippocampus in the diabetic rats and the presence of neuronal damage and death in the hippocampus and dentate gyrus in the experimental STZ-induced diabetes [23]. The effects of the antioxidative treatment support the hypothesis of an important role of oxidative stress and free radicals in neuronal pathology in diabetes and ischaemia. In our previous study, histological disorganization of the SN with increased NOS and TGF-*β*1 suggests that apoptosis begins as a result of oxidative stress in the diabetic SN [24]. These structural alterations during the progression of diabetic neuropathy also have been reported by other investigators [10]. Together with the results, it can be considered that especially in tissues containing neurons, the effects of oxidative stress occurs much more quickly and efficiently.

In conclusion, the presented results suggest that melatonin administration might be beneficial for reducing diabetic complications by preventing oxidative damage due to NOS increase in the diabetic rat. However, further molecular investigations are needed to elucidate the exact mechanism of action and to examine the potential therapeutic effects of MLT on diabetic tissue damage and apoptosis.
